# Recognition of vocoded words and sentences in quiet and multi-talker babble with children and adults

**DOI:** 10.1371/journal.pone.0244632

**Published:** 2020-12-29

**Authors:** Matthew J. Goupell, Garrison T. Draves, Ruth Y. Litovsky

**Affiliations:** 1 Department of Hearing and Speech Sciences, University of Maryland, Maryland, MD, United States of America; 2 Waisman Center, University of Wisconsin, Madison, WI, United States of America; 3 Department of Communication Sciences and Disorders, University of Wisconsin, Madison, WI, United States of America; University of Texas at Dallas, UNITED STATES

## Abstract

A vocoder is used to simulate cochlear-implant sound processing in normal-hearing listeners. Typically, there is rapid improvement in vocoded speech recognition, but it is unclear if the improvement rate differs across age groups and speech materials. Children (8–10 years) and young adults (18–26 years) were trained and tested over 2 days (4 hours) on recognition of eight-channel noise-vocoded words and sentences, in quiet and in the presence of multi-talker babble at signal-to-noise ratios of 0, +5, and +10 dB. Children achieved poorer performance than adults in all conditions, for both word and sentence recognition. With training, vocoded speech recognition improvement rates were not significantly different between children and adults, suggesting that improvement in learning how to process speech cues degraded via vocoding is absent of developmental differences across these age groups and types of speech materials. Furthermore, this result confirms that the acutely measured age difference in vocoded speech recognition persists after extended training.

## Introduction

Cochlear-implant (CI) users show substantial variability in speech recognition performance [1,2], a result of biological, surgical, and device-related factors [3]. To remove some of the unknown sources of variability in speech recognition performance, CI users’ performance can be studied using acoustic simulations of CI processing, a multi-channel vocoder, presented to normal-hearing (NH) listeners [4]. In a vocoder, an acoustic signal is bandpass filtered into a limited number of channels, the temporal envelope (i.e., the relatively slow amplitude variation over time of the acoustic waveform) is extracted from each channel, and these slowly varying envelopes are used to modulate a carrier signal such as a narrowband noise [5].

The way that the signal processing and CI simulation is performed, as well as other methodological choices, affect vocoded speech recognition performance. A larger number of vocoder channels increases performance because of improved spectral-temporal representation of the original acoustics [6–8]. The type of acoustic carrier affects performance because it affects the representation of the temporal envelope and the spectrum [9,10]. If frequency-to-tonotopic place mismatch is simulated, where speech information is now presented at higher frequencies than is typical (as would occur if the CI array has a relatively shallow insertion into the cochlea), an increase in shift would decrease performance [11,12]. In such a case, vowel formants would be shifted to higher frequencies and thus need to be adapted to or relearned. These signal processing choices may differentially affect the ability to perceive different speech materials such as consonants vs vowels and words vs sentences [6,10,13–15]. Other methodological approaches, such as if listeners receive training and how such training is implemented, will also affect performance [11,12,16–21]. Furthermore, these methodological choices may interact. For example, the use of a tonal carrier and a relatively small number (e.g., six) of channels introduces the need to consider the size of the auditory filters that contain stimulus energy. This is because the carrier sidebands caused by the envelope modulations might be resolved if the modulation rates are sufficiently high, and thus dramatically improve performance [9].

Characteristics about the listeners, such as their age and hearing status, will also affect performance. Towards the older end of the lifespan, hearing loss and/or advancing age decreases vocoded speech recognition [8,14,22,23]. Towards the younger end of the lifespan, vocoded speech recognition performance is often poorer for children compared to adults [12,13,24–29]. Studying vocoded speech recognition in children is important as it helps clarify how language is processed and learned via highly degraded signals as are presented via a CI. These studies have almost uniformly found that adults are better than children at vocoded speech recognition, although the exact age at which children reach adult-like performance has varied across studies. Eisenberg et al. [13] compared noise-vocoded speech recognition in children and adults. In that study, they tested two groups of children (ages 5–7 and 10–12 years) and adults (18–55 years), and used stimuli specifically designed for a child’s vocabulary (HINT-C, PBK, and VIDSPAC). There was no significant difference in vocoded speech recognition between the older children and adults when listening to speech processed into four or eight channels. However, vocoded speech recognition in the younger children was significantly worse than the older children and adults. Dorman et al. [26] found that children (3–5 years) needed more channels than adults on the easy and hard words of the multisyllabic lexical neighborhood test to achieve similar performance. Bertoncini et al. [30], tested children (5–7 years) and young adults on discrimination of 16-channel noise-vocoded vowel-consonant-vowel nonsense disyllables (i.e., a small closed set of stimuli), and found no significant difference between groups. Huyck [24] tested 11–13, 14–16, and 18–22 year old participants on meaningful English sentences with context using a six-channel noise vocoder and found that 11–13 year old listeners had worse performance than adults. As a whole, it appears that the exact age at which adult-like performance is achieved is impacted by the testing materials and procedure. In addition, there is evidence that non-sensory factors such as auditory attention and working memory may impact performance [31].

Another characteristic pertaining to the listeners that will affect performance is their previous exposure to vocoded speech because listeners adapt to the signal degradation and their scores improve with increased exposure and training. Adults with no prior exposure to vocoded speech can understand <10% of the words when listening to their first six-channel noise vocoded sentence, but quickly improve over a span of 20–30 sentences [18]. Training is critical when frequency-to-place mismatch or shift is introduced to the channel center frequencies. Experiments using shifted stimuli show large initial performance decrements and longer time scales of improvement until performance saturation compared to unshifted stimuli [11].

Extended training was omitted in many prior vocoded speech recognition studies with children; short testing periods are desirable when testing children because of attention and fatigue. For the previous studies that include training, there has been parallel improvement across groups of children and adults [12,24]. Such a finding is important; if vocoded speech recognition improvement with training differed between children and adults, this could introduce a confound for acute comparisons between groups. For example, children may improve more rapidly than adults in vocoded speech recognition to the point that the age-related differences are eliminated. Huyck [24] tested noise-vocoded sentences on different groups of adolescents and young adults (11–22 years); initial performance for all groups began at an average of >50% correct and improved approximately 5–10%. Waked et al. [12] tested eight-channel sine-vocoded matrix sentences using different amounts of shift on children (8–10 years) and young adults (19–23 years). For the 0- (control or no shift) and 3-mm (relatively small shift compared to the length of the CI array and typical 35-mm length of the cochlea) conditions, initial performance began at an average of >50% correct performance and improved approximately 20% after 4 hours of training. For the 6-mm (relatively large shift) condition, initial performance began at an average of approximately 25% correct and improved approximately 40% after 4 hours of training. Critically, the differences between adults and children disappeared for large 6-mm shift *and* low performance levels. Waked et al. [12] concluded that the lack of age-related performance differences between children and adults for the 6-mm shift condition was a result of the stimulus manipulation. An alternative interpretation, however, is possible; it was the initial performance level that produced the lack of age-related performance differences.

Therefore, we sought to clarify if shift or low performance caused the interaction between age, shift, and training seen in Waked et al. [12]. This was done by omitting shift as a factor, but testing materials that produced a range of performance. Specifically, we tested more difficult speech materials (words and sentences) compared to the closed set of matrix sentences used in Waked et al. [12], and included various levels of background noise. Across these conditions, we aimed to achieve a wide range of performance that would allow us to see if initial performance affects the differences in vocoded speech recognition between children and adults. This experiment was designed to answer three questions. (1) Do children and adults differ in vocoded word identification and sentence recognition? (2) Does training cause differential improvement in vocoded speech recognition between children and adults? (3) Are these performance differences and improvement rates affected by initial performance? We hypothesized that vocoded speech recognition would initially be worse for children than adults, children and adults would improve at different rates, the difference would be much smaller after training, and that there would be an interaction with condition such that age-related performance differences would not occur at the most difficult signal-to-noise ratios (SNRs).

## Materials and methods

### Listeners and equipment

Twenty children (8–10 years) and 21 young adults (19–26 years) were tested. All listeners had thresholds ≤20 dB Hearing Level between 0.25 and 8 kHz. All were native English-speaking with no reported developmental disabilities. None of the listeners had previous experience with listening to vocoded speech. All children assented and all adults consented to participation in the study, the process approved by the University of Wisconsin-Madison Institutional Review Board. The authors had no conflicts of interest and the Institutional Review Board oversaw the ethical conduct of the research.

The experiment was run using custom software in Matlab (The Mathworks, Natick, MA) and conducted in a standard double-walled sound booth with dimensions 7’ × 7’ × 6.5’ (IAC, New York, NY). The stimuli were delivered over circumaural headphones (HD650; Sennheiser, Hanover, Germany) driven by a real-time sound processor (RP2.1, PA5, and HB7; Tucker-Davis Technologies System 3, Alachula, FL).

### Stimuli

The stimuli consisted of target words and sentences spoken by different males, and both groups were presented the same stimulus corpuses. Only male talkers were necessary given that the stimuli would be vocoded [10]. The words were a closed set of 50 one-syllable, consonant-nucleus-consonant (CNC) words (list 1) [32]. The sentences were an open set of IEEE sentences [33], chosen from a list of 500 without replacement for each listener.

The male-spoken target speech was presented diotically at a level of 65 dB-A, either in quiet or in two-talker babble at one of three SNRs: 0, +5, and +10 dB. The root-mean-square energy over the entire target stimulus duration was used to calculate the resulting stimulus levels. The babble consisted of randomly selecting a portion of a string of IEEE sentences spoken by a single female, but two talkers were simulated by choosing different starting points in the continuous IEEE sentences. The babble was 5 s in duration for words and 8 s in duration for sentences, and the target word or sentence started 3 s after the beginning of the babble.

For the stimuli with babble, the male-spoken target and female-spoken babble were summed before vocoding. A pre-emphasis was applied by high-pass filtering the stimuli using a 1^st^-order Butterworth filter with a 1200-Hz cutoff frequency. The stimuli were then bandpass filtered into eight channels using 4^th^-order Butterworth filters, which provides a reasonable simulation of CI performance [6] and minimizes any effects of talker gender on performance [10]. The channel corner frequencies were logarithmically spaced between 300 and 8500 Hz. The envelope of each channel was extracted via half-wave rectification and low-pass filtering using a 2^nd^-order Butterworth filter with a 400-Hz cutoff frequency. The envelope of each channel was then used to modulate a narrowband noise carrier with a bandwidth that corresponded to the bandwidth of the filtered channel. The modulated noisebands were summed into an acoustic signal. For the conditions with the two-talker babble, ten tokens of target with masker were generated for each possible word or sentence for each SNR.

For the CNC words, the target was preceded by a preemptive word. There was an unprocessed 0.45-s word “Ready” spoken by a male talker, presented 1.55 s before the target word. If there was babble, “Ready” was spoken 1 s after the beginning of the babble. For sentences, there was no preemptive word before the target was presented.

### Procedure

#### Words

Ten children (age range = 8–10 years, mean = 8.6 years, standard deviation = 0.7 years) and 11 young adults (age range = 20–26 years, mean = 21.5 years, standard deviation = 1.8 years) were tested on a CNC word recognition task. Before testing, listeners were shown the written words, were asked to read each word out loud, and provide its definition. An experimenter corrected a listener’s pronunciation and/or provided the definition if necessary. For the children, a short pseudo-random test of the words was performed by the experimenter to ensure that the children could pronounce all words. All listeners correctly pronounced most of the words initially. Pronunciation and definitions were provided for only a small number of tokens and listeners. No listener was excluded based on their previous knowledge and their initial ability to correctly pronounce the words.

Adults were alone in the sound booth during testing, but children were accompanied by an experimenter to ensure that they could adequately control the computer user interface. The children could also ask the experimenter questions for clarification, if necessary.

Correct answer feedback appeared after each response, which was considered the training element. A single run consisted of 40 trials (10 words at each of the four SNRs). During each run, the words were picked randomly from the list with replacement. Different listeners were presented a different random order of words.

Both children and adults were tested on the same procedure in two separate two-hour sessions on different days. The second testing day was within three weeks of the first. All adults completed 20 runs; children completed 11–15 runs. Breaks were allowed as needed, typically about every three runs. The difference in the number of runs completed between was because the children needed longer and more frequent breaks than the adults [12].

#### Sentences

Ten different children (age range = 8–10 years, mean = 8.8 years, standard deviation = 0.8 years) and 10 different young adults (age range = 19–22 years, mean = 20.3 years, standard deviation = 0.9 years) were recruited for testing on IEEE sentence recognition. Unlike the CNC word recognition test, there was no familiarization with sentences. The method for testing with sentences was the same as that with words except that adult listeners typed their responses and an experimenter entered children’s responses (the experimenter verbally repeated the child’s response at an audible level, typed the response, and showed it to the child for approval). Correct-answer feedback was provided to all listeners. This was done visually for adults, and both visually and verbally for children. Each run consisted of 20 trials, five sentences presented at each of the four SNRs in randomized order. The sentences were randomly selected from the list of 500 sentences without replacement. All adults completed 20 runs; all children completed 15 runs.

## Results

**Fig 1:**
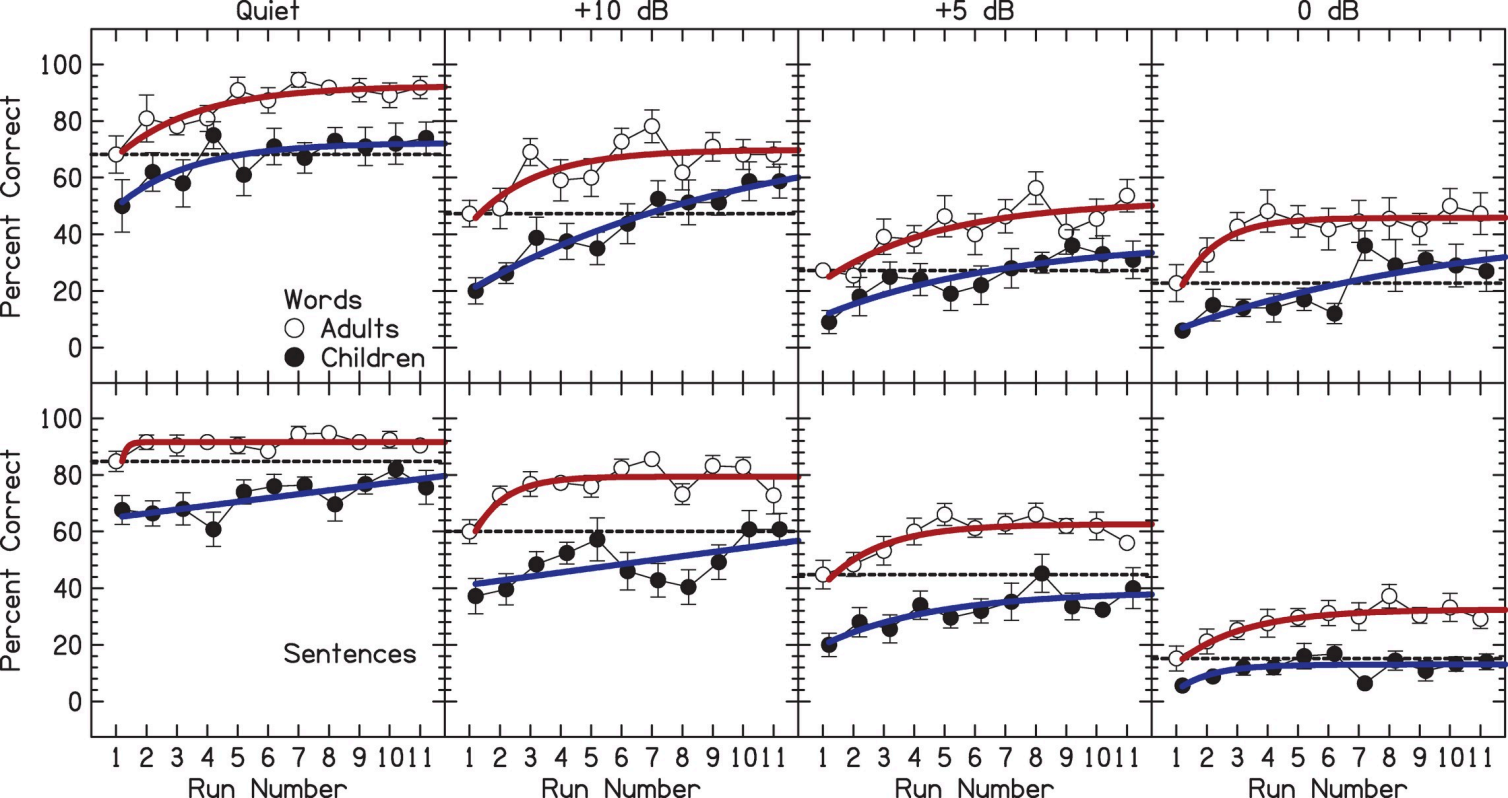
Percentage of correct responses as a function of run number. The error bars are ±1 standard error in length. Open symbols show adult performance and closed symbols child performance. The top row shows the results for words and bottom row for sentences. Solid lines represent fits to the data using a saturating exponential function, *y = A*[1−exp(−*Bx*)]+*C*. The horizontal dashed black line shows the average acute (run 1) performance for adults.

The percentage of correct responses was calculated for words and sentences. For words, percent correct for each condition and each run was based on ten words. For sentences, percent correct was determined from the percentage of key words that were correctly identified out of five key words per sentence [34]. The average percent correct as a function of run number is shown in Fig 1 for words (top panels) and sentences (bottom panels), at the four different SNRs. The percent correct values were transformed to rationalized arcsine units [35] and then were analyzed with a four-way mixed Analysis of Variance with factors between-subject factors of Group (child or adult) and Corpus (words or sentences), and within-subject factors of Run and SNR (quiet, 0, 5, or 10 dB). Only the first 11 runs were included in the analysis, the smallest number of runs completed by all of our listeners.

**Fig 2:**
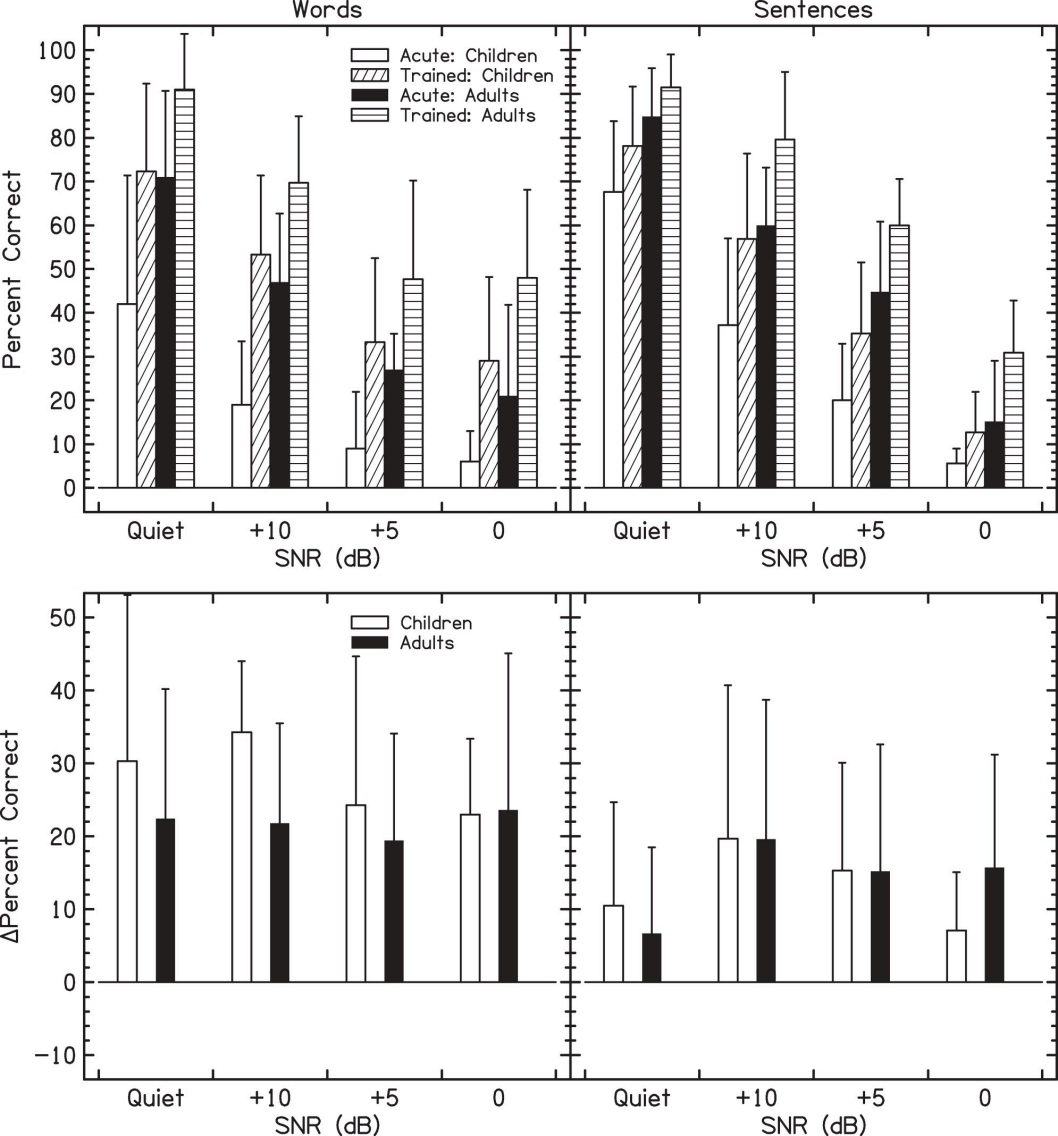
**The top row shows acute (run 1) vs. trained (average of runs 9–11) performance for words (left column) and sentences (right column).** The bottom row shows the difference between the trained and acute performance. Bars show the average and error bars show +1 standard deviation.

Adults performed better than children [F(1,37) = 48.4, *p*<0.0001]. There was no significant interaction with age (*p*>0.05 for all possible interactions), suggesting roughly parallel rates of improvement between adults and children for all conditions, including similar effects of SNR and of Corpus. This lack of interaction can also be seen in Fig 2, that compares acute (run 1) and trained (average of runs 9–11) performance.^1^ Performance significantly improved as a function of run number [F(10,370) = 29.4, *p*<0.0001]. This improvement statistically reached asymptote at run 7 (Helmert contrast: *p*<0.01 for up to run 6 vs. later, *p*>0.05 for all subsequent comparisons). More specifically, for words in quiet, adults had an average percent correct of 68% for run 1 (top row, left-most panel of Fig 1). Average percent correct increased and reached asymptote at run 7 at approximately 90%. Children had an average percent correct of 50% for run 1. Average percent correct increased and reached asymptote at about 70%. For sentences in quiet, adults had an average percent correct of 85% for run 1 (bottom row, left-most panel of Fig 1). Average percent correct increased and reached asymptote at approximately 90%. Children had an average percent correct of 68% for run 1. Average percent correct increased and reached asymptote at approximately 75%.

The data from each condition in Fig 1 was fit with a saturating exponential of the form:

y=A[1−exp(−Bx)]+C

where *x* is the run number, *y* is percent correct, and *A*, *B*, and *C* are free parameters. The acute performance for adults is also shown in Fig 1 with a horizontal black dashed line. In the top row for words, the fit lines for the child performance cross the black dashed line for acute adult performance between runs 5 and 7. In the bottom row for sentences, these lines never cross. The fits provide further evidence that adult performance was significantly better than child performance.

Reducing the SNR decreased performance [F(3,111) = 1225.2, *p*<0.0001] and each SNR was significantly different from the others (Tukey Honestly Significant Difference test: p<0.001 for all comparisons). The effect of corpus was not significant [F(1,37) = 1.56, *p* = 0.22]. There was a significant corpus×SNR interaction [F(3,111) = 56.3, *p*<0.0001], which occurred because performance for sentences was better than words at low SNRs, but worse than words at 0-dB SNR (see Fig 2). That is, context effects occurred in the sentences used in this study, whereby recognition of a portion of the sentence likely improved the probability of recognition other portions of the sentence. Therefore, listeners were disadvantaged at the 0-dB SNR for the sentences compared to the more favorable SNRs. Such context effects were absent for the isolated words. There was a significant corpus×run interaction [F(10,370) = 3.78, *p*<0.0001], which may have occurred because performance with sentences appeared to asymptote later in training than words.

## Discussion

This experiment was designed to investigate differences in child and adult vocoded speech recognition, particularly with regard to effects of training. We hypothesized that the difference in vocoded speech recognition between adults and children would be affected by the initial starting performance and training. While we found that adults were better than children at vocoded speech recognition, this difference was constant across conditions, and independent of starting performance level (Figs 1 and 2). Furthermore, with training, there was no differential improvement in vocoded speech recognition between adults and children. Taken together with the broader literature, it appears that differences in acute vocoded speech recognition scores across groups reflect those found for trained scores. Therefore, children demonstrate poorer vocoded speech recognition than adults across a wide range of ages, vocoder processing types, stimuli, and procedures [12,24]. Only cases that include frequency-to-place shift reduce differences between adults and children, where there is no difference by 6 mm of shift [12].

Although not directly addressed in the experimental design and analysis of the current study, it may be that the difference between children and adults in vocoded speech recognition is related to language development. A landmark study on vocoded speech recognition in children by Eisenberg et al. [13] showed that noise-vocoded speech recognition depended on whether stimuli were lexically easy or difficult, and differential use of context explained some of the differences across age groups. In another study, Nittrouer et al. [36] tested 40 native English-speaking adults and 7 year olds with four- and eight-channel noise-vocoded speech. They found that the native-speaking adults had the highest performance, followed by native-speaking children. They also tested 40 non-native English-speaking adults and found that their performance was worse than the native-speaking children, even though the non-native adults had a more mature auditory system. Those results suggest that the performance differences between children and adults are due to language development rather than maturity of the auditory system and processing of acoustical signals. The parallels between the current study and by Eisenberg et al. [13] are especially important given the stimuli differed greatly. Eisenberg et al. [13] used speech materials that were designed to be relatively child-friendly; the stimuli also controlled for word frequency and neighborhood density in the context of word recognition in isolation and when embedded in sentences. In the current study, we used a list of CNC words and IEEE sentences that are typically used with adult populations, which should have emphasized any performance differences that result from language abilities.

Non-sensory factors such as neurocognitive processes are another important aspect of developmental comparisons regarding vocoded speech recognition [13,18,37,38]. Recently Roman et al. [31] replicated Eisenberg et al. [13], but added several additional neurocognitive measures. The goal was to assess whether the ability to understand spectrally degraded speech was related to non-auditory abilities, namely auditory attention, talker discrimination, and verbal and non-verbal short-term working memory. There were significant correlations between measures of auditory attention, short-term memory, and the ability of children to understand isolated words and sentences that were vocoded. Given that the present study also used isolated words and sentences, there is an important conclusion to be drawn here. If neurocognition plays a role in impacting vocoded speech recognition, the age effects observed in the current study may be robust and related to maturation more so than to specific stimuli.

A different aspect of vocoded speech processing was assessed by Tinnemore et al. [39] who tested children’s ability to recognize emotional prosody in spectrally degraded stimuli. Here too there was a strong developmental effect during childhood, with adults outperforming children, and a strong predictor being non-verbal intelligence. The authors argue for the role of experience in modulating developmental changes in the ability of children to extract information from spectrally degraded speech.

Future directions for this work could address potential weaknesses of the current study. As there was no effect of initial performance, training over a wide range of conditions seems less important that focusing on a limited set of conditions so that the number of trials is larger to increase statistical power. While we chose speech materials typically aimed for adult populations, verification of potential differences with an unprocessed control condition would verify the age-related differences were not based on factors like vocabulary.

## Endnotes

The average of the last three runs was used to reduce variability for conditions where the performance had saturated and was stable; a similar approach could not be done for the first three runs because performance rapidly improved at the beginning of the experiment.

## Supporting information

S1 Table (XLSX)
